# Supplementary material for: Patterns of Intron Gain and Loss in Fungi
Source: PLoS Biol. 2004 Nov 30;2(12):e422. doi: 10.1371/journal.pbio.0020422 (PMC532390; doi:10.1371/journal.pbio.0020422)
Supplement: Table S1 — Also available at http://genes.mit.edu/NielsenEtAl/. (4.3 MB ZIP). [file pbio.0020422.st001.zip › NielsenEtAl/html/1313.html]

AN0174.1.NCU06558.1.MG08011.1.FG01730.1


```
 CLUSTAL W (1.82) Multiple Sequence Alignments - Introns Inserted


Sequence 1: MG08011.1	363 aa
Sequence 2: FG01730.1	361 aa
Sequence 3: AN0174.1	364 aa
Sequence 4: NCU06558.1	362 aa
Alignment Length: 371 aa
Number Identitical Residues: 136 aa
Alignment Score (without introns) 7283


MG08011.1 	-MP-KIVVLG2AGVSGLTAALLLSRNKDNDITVIAKHMPGDYDIEYASPWAGANVLP2MN
NCU06558.1	--MSTIVVVG2AGVSGLTCALQLAKQGGNTITVVAKHMPGDYDPEYTSPFAGANVLP~MA
FG01730.1 	-MSNTVVVIG2AGVIGLTSALLLAK-EGNSVTVVGKHMPGDSDAEYASPWAGANVVP2LA
AN0174.1  	MTKNNIVVLG2AGVSGLTTAYLLSQDPSNSVTVVAKHMPGDYDIEYCSPWAGANYLP2TG
          	   ..:**:* *** *** *  *::. .* :**:.****** * ** **:**** :*   

MG08011.1 	T--VEDSRWERRTFPELKRLAEEVPEAGIHLLK1VRLFRRTPASEDNETQTALDPIFAKD
NCU06558.1	P---EYNRWEGETWPELKRLAETCPEAGIHFQK~AVLYRRAQDEAAGFAGPLSDGLFVRN
FG01730.1 	P--KEASRWERRTWIALKKLVEETPEAGIHYQT1THILRRDSDMKSGKSGFSSN-FYADN
AN0174.1  	QPGTNHAKWERATWQPLKELTEKHPEAGIHFQE1SIVHNRKKDQET-TTGKWFSALVSEN
          	 .. :  :**  *:  **.*.*  ******      : .*        :    . :   :

MG08011.1 	PWYSEMFDD~FRTLSKDELPDGVVSANEFGSVCINTMLYLPWLVGQCRANGVVFNRANLT
NCU06558.1	PWYKDLVPD~YVDLPASEVPEGMSSASSFTSVCINTAIYLPWLVGQCRARGVVFKRAVLK
FG01730.1 	PWFKEIFNN~FRDNTPAELAAGYDSGFQYQGVCINVAIYLPWLLGQCLKYGVVVKRGILT
AN0174.1  	PWYKDVVPD0FKNLPKDQLAPGVDNAQVFTSVCINTAVYLPWLVGQCRKNGAVFKRAVFK
          	**:.::. : :   .  ::. *  ..  : .****. :*****:***   *.*.:*. :.

MG08011.1 	HIAEAAARHHSGAKADLVVNATGVTACKLGGVMDAAVQPARGQVVVVRNELPLMLGATLA
NCU06558.1	HISDAAKLSHTGRKPDIIINATGLLSCRLGGVMDKKVMPARGQVVLVRNEATPNMVCTSG
FG01730.1 	HINEAKYLSHTGKKANIIVNATGLGSLKLGGVEDTTVAPARGQVVVVRNETPKNMPLFMC
AN0174.1  	HVSDAANAHHSGQPADIVVNCTGLASKKLGGVRDDKLYPGRGQIVIVRNDPG-KMVSMSG
          	*: :*    *:*  .::::*.**: : :**** *  : *.***:*:***:    :     

MG08011.1 	AEDDAEHPDEMVYCMTRAAGGGTVIGGCYQKGNWDPNPDPNLAVRIMSRMVEFWPEIAD-
NCU06558.1	--TDDGNGDELCYIMQRAAGGGTILGGTYMKGNWDGVPDPNIATRIMKRAVEACPALTG-
FG01730.1 	SSTLDES-GEEIYAMQRAAGGGTVIGGTYQINNWDSQPCSNTANRIMQRIVDLMPEIAG-
AN0174.1  	--TDDGE-DELMYMMTRAAGGGTILGGCYQKHQWDPLPDPNLAVRIMKRAISICPELVGE
          	        .*  * * *******::** *   :**  * .* * ***.* :.  * :...

MG08011.1 	GKGVAGLDVIRHGVGLRPYREGGVRIEKERLSDGTWVVHNYGHAGWGYQGSYGCAERVVE
NCU06558.1	GKGIEALDVIRHAVGLRPYREGGVRIDKENING-TWVVHNYGHGGWGYQGSWGCAFRVQE
FG01730.1 	GKGITGLSIIRHGVGFRPYRKGGMRLEEEKLDDETWVVHNYGHSGWGYMASYGVAEGVVE
AN0174.1  	GQGIEGLDIIRHGVGLRPLRDDGPRIEAEMIEG-VAVVHNYGHGGFGYQASYGCAAEAVR
          	*:*: .*.:***.**:** *..* *:: * :.. . *******.*:** .*:* *  . .

MG08011.1 	LVDES---FKGKPKL
NCU06558.1	LVDEIKSELKLGSKL
FG01730.1 	LVAKV---TGTRAKL
AN0174.1  	LVKDT-LQKKIRAKL
          	** .   .    .**
```
